# Midwife-led maternity care in Ireland – a retrospective cohort study

**DOI:** 10.1186/s12884-017-1285-9

**Published:** 2017-03-28

**Authors:** Anna Dencker, Valerie Smith, Colette McCann, Cecily Begley

**Affiliations:** 1 Gothenburg Centre for Person-Centred Care (GPCC), Sahlgrenska Academy, University of Gothenburg, Box 457, 405 30 Gothenburg, Sweden; 2 Institute of Health and Care Sciences, Sahlgrenska Academy, University of Gothenburg, Gothenburg, Sweden; 3 School of Nursing and Midwifery, Trinity College Dublin, Dublin, D02T283 Ireland; 4 Our Lady of Lourdes Hospital, Drogheda, Ireland

**Keywords:** Midwife-led care, Childbirth, Low-risk pregnancy, MidU study

## Abstract

**Background:**

Midwife-led maternity care is shown to be safe for women with low-risk during pregnancy. In Ireland, two midwife-led units (MLUs) were introduced in 2004 when a randomised controlled trial (the MidU study) was performed to compare MLU care with consultant-led care (CLU). Following study completion the two MLUs have remained as a maternity care option in Ireland. The aim of this study was to evaluate maternal and neonatal outcomes and transfer rates during six years in the larger of the MLU sites.

**Methods:**

MLU data for the six years 2008–2013 were retrospectively analysed, following ethical approval. Rates of transfer, reasons for transfer, mode of birth, and maternal and fetal outcomes were assessed. Linear-by-Linear Association trend analysis was used for categorical data to evaluate trends over the years and one-way ANOVA was used when comparing continuous variables.

**Results:**

During the study period, 3,884 women were registered at the MLU. The antenatal transfer rate was 37.4% and 2,410 women came to labour in the MLU. Throughout labour and birth, 567 women (14.6%) transferred to the CLU, of which 23 were transferred after birth due to need for suturing or postpartum hemorrhage. The most common reasons for intrapartum transfer were meconium stained liquor/abnormal fetal heart rate (30.3%), delayed labour progress in first or second stage (24.9%) and woman’s wish for epidural analgesia (15.1%). Of the 1,903 babies born in the MLU, 1,878 (98.7%) were spontaneous vaginal births and 25 (1.3%) were instrumental (ventouse/forceps). Only 25 babies (1.3%) were admitted to neonatal intensive care unit.

All spontaneous vaginal births from the MLU registered population, occurring in the study period in both the MLU and CLU settings (*n* = 2,785), were compared. In the MLU more often 1–2 midwives (90.9% vs 69.7%) cared for the women during birth, more women had three vaginal examinations or fewer (93.6% vs 79.9%) and gave birth in an upright position (standing, squatting or kneeling) (52.0% vs 9.4%), fewer women had an amniotomy (5.9% vs 25.9%) or episiotomy (3.4% vs 9.7%) and more women had a physiological management of third stage of labour (50.9% vs 4.6%).

**Conclusions:**

Midwife-led care is a safe option that could be offered to a large proportion of healthy pregnant women. With strict transfer criteria there are very few complications during labour and birth. Maternity units without the option of MLU care should consider its introduction.

## Background

Midwifery-led units, designed for women who prefer little or no medical intervention, have been shown to decrease the risk of interventions during labour and birth and increase spontaneous vaginal births and maternal satisfaction [1, 2]. Midwife-led models of care include continuous support, continuity of care provider and a home-like environment for women with low risk during pregnancy and childbirth [3, 4], and there is good scientific evidence that continuous support during labour and birth offers several advantages and no adverse effects [5–7]. A framework mixed-methods analysis identified reasons as to why midwifery care during pregnancy and childbirth is beneficial, including; midwifery care supports normality in pregnancy and childbirth, focuses on prevention and support, stresses respectful relationships, and is cost-effective [8–10]. In addition, midwife-led models include continuity of childbirth care, which is also important for women [11]. Furthermore, women should have the option to decide place of birth and choose between hospital care and midwife-led care [12].

In Ireland, two alongside Midwife-led units (MLU) were introduced in 2004 and evaluated in a randomised controlled trial (RCT) 2004–2007, the MidU trial (ISRCTN14973283) [13]. Pregnant low-risk nulliparous and parous women were included. Those allocated to MLU received pregnancy, childbirth and postnatal care by the midwives in a homelike environment at the ‘alongside’ MLU, situated on a floor of the parent maternity unit. They were transferred to the consultant-led unit (CLU) nearby if necessary and at any stage. The results showed that MLU care was as safe as consultant-led care that was provided to the control group [13] but was associated with fewer interventions, like having continuous electronic fetal monitoring or augmentation of labour. Midwife-led care also cost €182 less per woman [14], and resulted in greater satisfaction for some aspects of care [15].

The MLU units have continued to operate successfully after the trial and the aim of this study was to evaluate maternal and neonatal outcomes and transfer rates during the succeeding 6 years in the larger of the two MLU sites. A second aim was to study interventions rates (amniotomy, episiotomy), care variables (number of carers, number of vaginal examinations, upright position for birth, physiological management of third stage of labour) in women opting for MLU care.

## Methods

### Design, setting and participants

A retrospective cohort study, of all women who were low-risk for maternity complications, and who opted for midwife-led pregnancy and childbirth care at the MLU study site during 2008 to 2013, was conducted, using hospital register data. These data, following permissions, were accessible on the hospital’s Maternity Information System (MIS). All information on the MIS is contemporaneously entered, by a midwife, or other hospital staff member, as women make contact with the maternity service (e.g. booking history, any antenatal admission(s), labour and birth admission and postnatal stay). The information on the MIS includes, also, specific birth and neonatal outcome details (e.g. mode of birth, live birth, admission to neonatal unit, etc,).

The MLU site, in this study, is situated as an alongside, small unit within Our Lady of Lourdes Hospital, Drogheda, Ireland (4,000 births per year). Women opting for MLU care register before 24 weeks in pregnancy. In MLU a small group of midwives provide care during pregnancy, intrapartum, and postpartum and the women will receive care from any midwife in the group. When/if complications/risk factors arise, the woman is transferred to the CLU temporarily or permanently. Criteria for transfer are agreed on, and include complications such as hypertension in pregnancy, antepartum haemorrhage, induction of labour and meconium-stained liquor, for example. In the MLU, the women/families receive postpartum care for 1–2 days, but most choose to return home on the first day after birth. MLU midwives visit a postpartum woman, at home, on her first day following discharge from the MLU, and again, as necessary, over the course of the following week. Most women receive two postnatal home visits.

The study sample was women who opted for MLU care, some of whom were transferred to CLU in pregnancy or labour. All spontaneous vaginal births from this population, occurring in the study period in both the MLU and the CLU settings (*n* = 2,778), were analysed in order to compare MLU and CLU interventions, support of normality in labour and birth and key clinical outcomes. Included in the CLU group were some spontaneous births that occurred in the theatre and outside the labour ward (*n* = 32).

### Data collection

Obstetric characteristics and maternal and neonatal outcomes, such as, mode of birth, acceleration of spontaneous labour and neonatal Apgar scores, on all women, were recorded in their charts and in the hospital MIS. Following research ethics approval from the university and hospital site, data were abstracted from this database. Any apparent anomalies on the MIS were queried and cross-checked with the written details in the individual woman’s medical chart.

### Data analyses

Linear-by-Linear Association trend analysis was used in the analyses of categorical data. One-way ANOVA was used for comparing continuous variables. Data were analysed with SPSS 23 for Windows (SPSS Inc., Chicago, IL, USA) and *p*-values of <0.05 were considered significant.

## Results

**Fig. 1:**
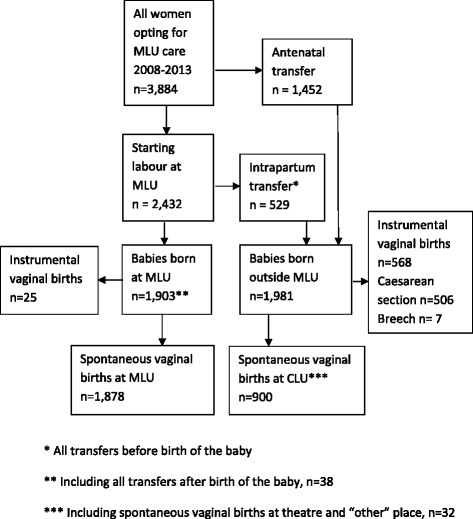
Flow diagram of study population

In total, 3,884 women, of low risk for pregnancy and childbirth complications, opted for MLU care during the years 2008–2013. A flow diagram of the study population is shown in Fig. 1. During the study period the proportion of women who actually gave birth to their baby at the MLU was 49.0%. The mean maternal age was 30.0 years. The proportion of women in the study sample who had their labour induced, which is performed only in the CLU, increased from 15.8% in 2008 to 20.4% in 2013 (*p* = 0.016). Oxytocin treatment to accelerate spontaneous labour, and the rates of spontaneous vaginal and instrumental vaginal births, all remained stable over the years (11.5%, 71.7% and 15.3%, respectively). The rate of caesarean section increased slightly, from 12.0% to 14.8% (*p* = 0.047), which is in line with national trends. The proportion of nulliparous women opting for MLU care more than doubled during the study period, from 16.0% to 38.9% (*p* < 0.001). Characteristics and childbirth outcomes for the whole group are shown in Table 1.

**Table 1:** Obstetric characteristics and outcomes for women opting for MLU care from 2008 to 2013, *n* = 3,884

	Year 2008	Year 2009	Year 2010	Year 2011	Year 2012	Year 2013	Total	*p*-value
	*n* = 594	*n* = 564	*n* = 668	*n* = 631	*n* = 705	*n* = 722	*n* = 3,884	
Nulliparous women, %	16.3	19.3	25.1	35.8	46.5	38.9	31.1	<0.001 ^a^
Maternal age, years	29.9	30.0	29.9	30.0	29.9	30.2	30.0	0.480 ^b^
Gave birth at MLU, %	45.6	50.0	52.4	49.8	49.2	47.0	49.0	0.972 ^a^
No antenatal transfer, %	50.5	66.1	68.3	66.1	63.5	60.7	62.6	0.022 ^a^
Induction of labour, %	15.8	18.8	18.4	22.2	20.7	20.4	19.5	0.016 ^a^
Acceleration of spontaneous labour with oxytocin, %	13.2	11.2	12.4	9.3	10.8	12.1	11.5	0.451 ^a^
Spontaneous vaginal birth, %	73.2	73.0	72.9	71.2	71.6	68.8	71.7	0.054 ^a^
Instrumental vaginal birth, %	14.8	14.2	15.9	16.5	13.8	16.3	15.3	0.580 ^a^
Caesarean section, %	12.0	12.8	11.2	12.4	14.6	14.8	13.0	0.047 ^a^
PPH over 500 mL, %	12.9	14.4	13.6	13.5	16.1	15.7	14.4	0.095 ^a^
PPH over 1000 mL, %	5.0	5.6	5.1	5.6	5.1	4.9	5.2	0.795 ^a^
Apgar Score <7 at 5 min, %	0.7	0.2	0	0.5	0.4	0.3	0.3	0.720 ^a^

^a^ Linear-by-Linear Association

^b^ One-way ANOVA

The proportion of women that were not transferred from the MLU to the CLU averaged 48.0% for the whole group over the years (*p* = 0.562). Fewer women were transferred antenatally, and antenatal transfers decreased significantly from 49.5% to 39.3% (*p* = 0.022). Intrapartum transfers increased for both primiparous and multiparous women, and for the total group from 4.5% to 14.0%. The proportion of women transferred postpartum for suturing was very small (*n* = 23 women; 0.6%). The most common reasons for intrapartum transfers were meconium stained liquor (31.4%), delayed labour progress in the first or second stage of labour (25.9%) and a woman’s wish for epidural analgesia (14.5%), which was only available in the CLU environment. Transfer rates are shown in Table 2, with reasons for transfers presented in Table 3.

**Table 2:** Transfer rates for women opting for MLU care from 2008 to 2013 by timepoint for transfer parity, and year, *n* = 3,884

Transfer timepoint	Year 2008	Year 2009	Year 2010	Year 2011	Year 2012	Year 2013	Total *n* = 3,884	*p*-value ^a^
	*n* = 594	*n* = 564	*n* = 668	*n* = 631	*n* = 705	*n* = 722		
Antenatal transfer, %	49.5	33.9	31.7	33.9	36.5	39.3	37.4	0.022
*Primiparous women*	59.8	46.8	46.4	41.6	46.6	51.4	47.8	
*Multiparous women*	47.5	30.8	26.8	29.6	27.6	31.5	32.6	
Intrapartum transfer, %	4.5	16.0	17.1	16.0	15.7	14.0	14.0	<0.001
*Primiparous women*	9.3	25.7	25.0	27.0	22.6	22.1	22.8	
*Multiparous women*	3.6	13.6	14.4	9.9	9.8	8.8	10.0	
Postpartum transfer, %	0	0.5	1.0	0.2	1.4	0.3	0.6	0. 247
*Primiparous women*	0	0	1.2	0	1.2	0.4	0.6	
*Multiparous women*	0	0.7	1.0	0.2	1.6	0.2	0.6	
No transfer, %	46.0	49.6	50.1	49.9	46.4	46.5	48.0	0. 562
*Primiparous women*	30.9	27.5	27.4	31.4	29.6	26.1	28.7	
*Multiparous women*	48.9	54.9	57.8	60.2	61.0	59.4	56.7	

^a^ Linear-by-Linear Association

**Table 3:** Reasons for transferral intra- and postpartum, *n* = 567^a^

Reason for transfer	(%)
Fetal reason including meconium stained liquor	31.4
Delayed labour progress in first or second stage	25.9
Woman’s wish for epidural analgesia	14.5
Preterm pregnancy	7.9
Breech/malpresentation	1.2
PPH or/and retained placenta	6.5
MLU at capacity	6.3
Suturing	3.7
Miscellaneous	2.5

^a^ Number of transfers before birth of the baby was 529, after birth 38

During the study period 1,903 women (49.0%), of all women opting for MLU care, gave birth at the MLU, including 24 women who underwent ventouse-assisted birth and 1 woman who underwent forceps-assisted birth. A majority of these 1,903 women (90.7%) had one or two carers (midwives), only, during labour and birth. Approximately half (51.3%) gave birth in an upright position; that is, standing, squatting or kneeling, and rates of episiotomy (3.8%) and sphincter ruptures (1.6%) were low. The numbers of women with an intact perineum after birth were stable over the years (*p* = 0.687) at a mean rate of 40.0%. Rates of amniotomy varied significantly (*p* = 0.001) during the study period, from between 2.2% at their lowest to 8.7% at their highest. In 50.3% of women, the third stage of labour was physiologically managed. Postpartum haemorrhage (PPH) over 500mls increased slightly (*p* = 0.012) but was low, overall, at 3.0% during the study period. PPH over 1,000mls did not increase (*p* = 0.941) and was 1.3% overall. Very few babies (0.1%) were born with an Apgar score below 7 at 5 min (Table 4).

**Table 4:** Obstetric data and care variables of all births at MLU year 2008 to 2013, *n* = 1,903

	Year 2008	Year 2009	Year 2010	Year 2011	Year 2012	Year 2013	Total *n* = 1,903	*p*-value ^a^
	*n* = 271	*n* = 282	*n* = 350	*n* = 314	*n* = 347	*n* = 339		
1-2 midwives involved, %	87.1	94.7	91.2	88.9	90.8	91.1	90.7	0. 715
Upright position, %	48.7	49.3	50.6	62.1	53.6	43.7	51.3	0. 814
Amniotomy, %	3.4	2.2	5.3	8.7	7.5	7.2	5.9	0.001
Spontaneous vaginal birth, %	98.9	99.3	98.9	98.1	99.1	97.9	98.7	0.271
Intact perineum, %	41.6	44.6	34.8	39.8	39.4	41.1	40.0	0. 687
Episiotomy, %	2.6	2.8	4.3	5.4	3.2	4.4	3.8	0. 257
Sphincter tear, %	0.7	1.1	2.3	1.0	2.3	2.1	1.6	0. 147
Physiological management of 3^rd^ stage, %	29.9	42.6	45.7	56.7	58.5	63.7	50.3	<0.001
PPH > 500 mL, %	2.2	1.1	2.6	2.5	5.2	3.8	3.0	0. 012
PPH > 1000 mL, %	1.9	0.4	0.9	1.3	2.9	0.3	1.3	0. 941
Apgar score <7 at 5 min, %	0.4	0	0	0	0	0	0.1	0. 117

^a^ Linear-by-Linear Association

Comparisons between spontaneous vaginal births at the MLU and spontaneous vaginal births in women opting for MLU who were subsequently transferred to CLU care are shown in Table 5. In the CLU population spontaneous births in the theatre and outside the labour ward (*n* = 32) are included. In the group of women who remained in the MLU for all their labour and birth, the proportion of primiparous women were lower (*p* < 0.001) and the women were older (*p* < 0.001) than those who transferred to the CLU. In the MLU fewer midwives (*p* < 0.001) cared for the women during birth, more women had three vaginal examinations or fewer, and gave birth in an upright position, i.e. standing, squatting or kneeling (*p* < 0.001), fewer women had an amniotomy (*p* > 0.001), fewer women had an episiotomy (*p* > 0.001) and more women had a physiological management of the third stage of labour (Table 5).

**Table 5:** Comparison of intervention rates, care variables and key outcomes in the study group (all women opting for MLU care), between all spontaneous vaginal births at MLU vs CLU

	MLU	CLU	*p*-value
	*n* = 1,878	*n* = 900*	
Primiparity, %	18.8	25.9	<0.001 ^a^
Maternal age, years	30.6	29.5	<0.001 ^b^
*Primiparous women*	27.4	26.6	
*Multiparous women*	31.4	30.6	
1-2 midwives involved, %	90.9	70.0	<0.001 ^a^
*Primiparous women*	88.4	68.3	
*Multiparous women*	91.5	70.6	
Maximum 3 vaginal examinations during birth, %	93.6	79.7	<0.001 ^a^
*Primiparous women*	86.8	69.4	
*Multiparous women*	95.1	83.2	
Upright position, %	52.0	9.4	<0.001 ^a^
*Primiparous women*	52.0	6.9	
*Multiparous women*	52.0	10.3	
Amniotomy, %	5.9	25.9	<0.001 ^a^
*Primiparous women*	5.5	24.0	
*Multiparous women*	6,1	26.6	
Episiotomy, %	3.4	9.4	<0.001 ^a^
*Primiparous women*	7.3	22.7	
*Multiparous women*	2.4	4.8	
Sphincter tear, %	1.7	2.0	0.539 ^a^
*Primiparous women*	4.2	3.4	
*Multiparous women*	1.0	1.5	
Physiological management of 3^rd^ stage, %	50.9	4.7	<0.001 ^a^
*Primiparous women*	50.8	2.1	
*Multiparous women*	50.9	5.5	
PPH > 500 mL, %	2.9	7.2	<0.001 ^a^
*Primiparous women*	5.1	7.7	
*Multiparous women*	2.4	7.0	
PPH > 1000 mL, %	1.3	2.8	0.008 ^a^
*Primiparous women*	2.6	3.4	
*Multiparous women*	1.0	2.6	
Apgar score <7 at 5 min, %	0.1	0.5	0.038 ^a^
*Primiparous women*	0.0	0.9	
*Multiparous women*	0.1	0.3	

^a^ Fisher’s Exact Test

^b^ One-way ANOVA

* Including spontaneous vaginal births at theatre and “other” place, *n* = 32

## Discussion

This 6-year follow-up study, post the MidU Trial [14] (2008–2013), and 10 years since the introduction of MLUs in Ireland, demonstrates ongoing support for midwife-led care as a safe and viable option for healthy, low-risk pregnant women. During the study period, the antenatal transfer rate, from the MLU to the CLU, decreased and more women who opted for MLU care started their labour at the MLU. Rates of instrumental births remained low, rates of interventions and complications were also low, and very few babies needed neonatal intensive care. Births at the MLU were characterised by a high number of women giving birth in upright positions, having few carers (greater continuity of care), experiencing few vaginal examinations, and having the third stage of labour managed physiologically. Other interventions, such as amniotomy and episiotomy, also decreased over time. Collectively, these results reinforce the results of international literature that suggests midwife-led childbirth care is a safe option that supports normality in labour and birth.

Although the transfer rate, overall, was stable over the years, the decreasing antenatal transfer rates suggest that more women were enabled to start labouring at the MLU and, instead, were transferred during labour to the CLU as needed. The greater numbers of nulliparous women requiring intrapartum transfers, might be reflective of the greater number of nulliparous women accessing the MLU over the years, rather than ‘true’ increases, although, intrapartum transfers also increased in multiparous women (Table 2). Intrapartum transfer is often a sub-optimal/negative experience for women [11] causing maternal anxiety. However, the MLU and the CLU are situated in the same building and belong to the same care organisation. This leads to an easier transfer during labour for most women, and transfers are managed with limited inconvenience. Increases in the intrapartum transfer rates over the study period, may reflect, in part, overall increasing intervention in childbirth, for example, increasing rates of induction and acceleration of labour [16, 17], in maternity care, in general.

For the group of women who remained in the MLU throughout pregnancy and birth, the maternal and neonatal outcomes showed low rates of sphincter tears, high rate of intact perineum, low rate of PPH, and very few babies with low Apgar scores, in accordance with previous research [1, 18]. Furthermore, these women had very good outcomes in terms of the care they received, such as few carers, few vaginal examinations during labour, and increased physiological management of the third stage of labour. Therefore, for these women with low risk, MLU care seemed to reduce the risk of unnecessary interventions.

Interventions such as amniotomy and episiotomy are often used routinely during birth without showing positive outcomes [19, 20]. These interventions, when compared in women who had spontaneous vaginal births at the MLU and women who had spontaneous vaginal births at the CLU, were found to be much more common in the CLU, corroborating earlier research [5, 12, 17, 18, 21]. Some of these differences might be explained by risk factors occurring during labour and birth but do not explain the large differences in both primiparous and multiparous women (Table 5). The most common reason for intrapartum transfer was meconium-stained liquor and/or fetal heart rate abnormalities, which would not necessarily affect the amniotomy and episiotomy rates, nor should they affect the rate of physiological management of the third stage of labour. It is thus likely, that the difference in rate of interventions in women with spontaneous vaginal birth is due to different care philosophies in the different units [22]. The increased rate of interventions in the CLU did not seem to result in important differences in outcomes such as PPH, Apgar score less than 7 and sphincter tear rates, which were similar across the units or slightly lower, only, in the MLU births. The reduced rates of PPH in the MLU are interesting as half of all women in MLU had a physiological management of the third stage, in line with recommendations for low risk women [23].

Quality in childbirth care [10] can be examined with care variables such as maternal position for birth [24], number of carers and number of vaginal examinations. All of these care indicators were lower in the MLU, showing that providing care in MLUs is one way to improve the quality of childbirth care for healthy, low risk women [2, 5]. Being able to choose a comfortable position may influence the birth experience for women and using a variety of birth positions indicates that birthing women have more influence and control over their births [24] and may explain why women are more satisfied with midwife-led care [1, 2, 5, 15]. A low rate of vaginal examinations could also be used as a quality of care indicator because they are often experienced as uncomfortable and do not benefit the progress of labour [25].

It has taken a long time for Ireland to reach acceptance, and permit the introduction, of midwife-led care [26]. However, given the continued positive outcomes of midwife-led care demonstrated here, in addition to the successful and cost-effective outcomes of the previous trial [14, 15], and international literature [2], it is clear that midwife-led care should now be extended to other units in the country. The recent Maternity Strategy, launched in 2016 [27] defines a supported care pathway, as one “intended for normal-risk mothers and babies, with midwives leading and delivering care within a multidisciplinary framework”. Thus, there is no barrier to the roll-out of birth centres led by midwives, across Ireland.

### Strengths and limitations

Limitations of this study include the retrospective design, and, the potential influence of factors, other than those reported, on care during labour and birth in the compared groups who had a spontaneous vaginal birth at MLU and CLU. In the group that birthed spontaneously at the CLU, (having transferred from the MLU), some would have been transferred from MLU care due to risk factors and some only because the MLU was at capacity. These data, therefore, are not comparable with the group that birthed spontaneously at the MLU, especially women who were transferred due to prolonged labour who would have had interventions at the CLU. However, episiotomy, maternal position for birth, number of carers, number of vaginal examinations, and physiological management of third stage are not always directly influenced by obstetric risk factors.

The study sample included in this study are representative of the study’s target population (that is, all low risk women attending the two MLUs in Ireland during the study period) because the MLU chosen for this study was larger than the second MLU by approximately 2.5 times in terms of the number of births per annum and the numbers attending the MLUs on a monthly or annual basis. For this reason, we are confident that the study sample size was sufficiently large to allow for the results to be generalised and for inferences to be made to the wider target population.

## Conclusions

Ten years following the introduction of MLUs in Ireland, midwife-led care remains demonstrably a safe option that could be offered to a large proportion of healthy pregnant women. With strict transfer criteria there are very few complications during labour and birth, and outcomes remain good. Maternity units without the option of MLU care, in both Ireland and across the world, should consider its introduction.

## Abbreviations

CLU: Consultant-led unit
MIS: Maternity Information System
MLU: Midwifery-led unit
PPH: Postpartum haemorrhage
